# Nutritional Strategies for Methane, Nitrogen, and Phosphorus Mitigation in Ruminants: Mechanisms, Applications, and Regional Adaptations

**DOI:** 10.3390/microorganisms14071410

**Published:** 2026-06-26

**Authors:** Zhenming Wang, Mingjin Wang, Hongren Su, Jingyi Shi, Sifan Dai, Ruiyun Zhang, Dongwang Wu

**Affiliations:** College of Animal Science and Technology, Yunnan Agricultural University, Kunming 650201, China; wangzm369@126.com (Z.W.); 17387334493@163.com (M.W.);

**Keywords:** ruminants, green nutrition, methane mitigation, hydrogen metabolism, rumen microbiota, nitrogen and phosphorus excretion, precision nutrition, multi-omics

## Abstract

Against the background of the ongoing transition toward green livestock production and the implementation of “dual-carbon” goals, reducing the environmental footprint of ruminant production while maintaining animal performance has become a key focus in nutritional regulation research. This review summarizes and critically discusses the mechanisms and recent progress of nutritional strategies for emission mitigation and improved nutrient utilization in ruminants, with particular emphasis on major environmental challenges, including enteric methane emissions and nitrogen and phosphorus losses. The review discusses the underlying mechanisms through which nutritional interventions contribute to emission reduction, focusing on ruminal hydrogen flux allocation, microbial community remodeling, host metabolic responses, and changes in nutrient utilization efficiency. In addition, by integrating functional feed additives, diet formulation optimization, precision protein supply, and mineral nutrition regulation, this review compares the application characteristics and practical challenges of different strategies in mitigating methane emissions, reducing nutrient excretion, and sustaining production performance. Furthermore, research approaches such as in vitro screening, multi-omics analysis, and evidence synthesis approaches are discussed to highlight the shift of green nutritional technologies from single-target interventions toward integrated and system-level regulation. This review provides a theoretical basis and technical reference for developing green ruminant production systems that balance productivity, animal health, and environmental sustainability.

## 1. Introduction

With persistent global food security pressures and increasing competition for feed grains [1], ruminant production plays a critical role in global agriculture, particularly in the context of the transition toward green livestock systems and the implementation of “dual carbon” goals [2]. Owing to their ability to efficiently utilize roughage and non-grain feed resources, ruminants contribute substantially to alleviating feed–food competition, improving the utilization of grassland resources, and ensuring the stable supply of animal-derived products [3,4]. However, the unique process of ruminal fermentation is also associated with methane emissions and nitrogen and phosphorus losses, making ruminant production an important contributor to agricultural environmental burdens [5,6,7]. Therefore, reducing environmental impacts and improving resource-use efficiency while maintaining animal performance and health have become key objectives in research on ruminant nutritional regulation.

In recent years, considerable progress has been made in nutritional strategies aimed at reducing the environmental impacts of ruminant production. Current research focuses on feed additives, dietary optimization, nutrient supply improvements, and manure management, which have contributed to methane mitigation and nutrient loss control to varying degrees [8,9,10,11]. Nevertheless, several challenges remain, including an incomplete understanding of the underlying mechanisms, large variations in the effectiveness of different strategies, insufficient long-term stability, and unclear trade-offs or synergies between production performance and environmental benefits [5,12,13,14]. Therefore, this review summarizes recent advances in green nutritional regulation and emission mitigation in ruminants, with the aim of providing theoretical support and practical guidance for future research and sustainable ruminant production. Previous reviews have provided valuable syntheses of enteric methane mitigation strategies, the mechanisms and efficacy of anti-methanogenic compounds, and the quantitative effects of specific additives such as 3-nitrooxypropanol [15,16,17]. Building on these important contributions, the present review seeks to provide a complementary perspective by considering methane mitigation together with nitrogen and phosphorus excretion control within a broader green nutrition framework. In particular, this review links ruminal hydrogen metabolism and microbial community remodeling with precision nutrient supply, species-specific responses, regional suitability, and methodological evaluation from in vitro screening to system-level environmental assessment. By emphasizing both biological mechanisms and practical application boundaries, this review aims to support the development of nutritional strategies that are not only effective in reducing emissions but also compatible with animal performance, nutrient-use efficiency, and regional production conditions.

To improve the transparency of this review, a literature search was conducted using major scientific databases, including Web of Science, PubMed, Scopus, and Google Scholar. The literature search covered publications available up to the final literature update in 2026. The search terms included combinations of “ruminants,” “green nutrition,” “methane mitigation,” “enteric methane,” “rumen microbiota,” “hydrogen metabolism,” “3-nitrooxypropanol,” “tannins,” “saponins,” “nitrogen excretion,” “phosphorus excretion,” “precision nutrition,” “multi-omics,” and “life cycle assessment.” Peer-reviewed articles, review papers, and meta-analyses related to nutritional regulation, rumen fermentation, methane mitigation, and nitrogen and phosphorus loss reduction in ruminants were considered. Studies were prioritized when they provided mechanistic evidence, in vivo validation, systematic reviews, or quantitative comparisons across dietary strategies, additives, animal species, or production systems. Articles not directly related to ruminant nutrition, emission mitigation, nutrient utilization, or environmental assessment were excluded.

## 2. Mechanisms and Nutritional Strategies for Mitigating Methane Emissions from Ruminants

Methane emissions from ruminants primarily originate from the metabolic activity of methanogenic archaea during anaerobic rumen fermentation. This process essentially involves the reduction of CO_2_ to CH_4_ by methanogenic archaea using H_2_ as an electron donor [18,19]. These archaea are mainly affiliated with the genus Methanobrevibacter, which represents the dominant methanogenic group in the rumen of ruminants [18,20]. The abundance of methanogens, such as *M. gottschalkii*, *M. smithii*, *M. boviskoreani*, *M. millerae*, and *M. thaueri*, is positively correlated with methane emissions [18,21]. However, methane production is not driven by a single microbial group; rather, it is jointly regulated by microbial interactions, hydrogen flow distribution, and dietary substrate supply within the rumen [7,18,19].

During rumen fermentation, bacteria, fungi, and protozoa degrade carbohydrates to produce volatile fatty acids (VFAs), CO_2_, and H_2_ [22]. If excess H_2_ is not removed in a timely manner, it can thermodynamically inhibit further fermentation by increasing hydrogen partial pressure and impairing the reoxidation of NADH [7]. Methanogenic archaea use H_2_ as an electron donor and CO_2_ as an electron acceptor to generate CH_4_, thereby maintaining a low hydrogen partial pressure and supporting rumen fermentation homeostasis [23]. Therefore, methane production not only reflects the metabolic activity of methanogens but also indicates changes in hydrogen flow distribution and microbial ecological networks within the rumen.

**Figure 1:**
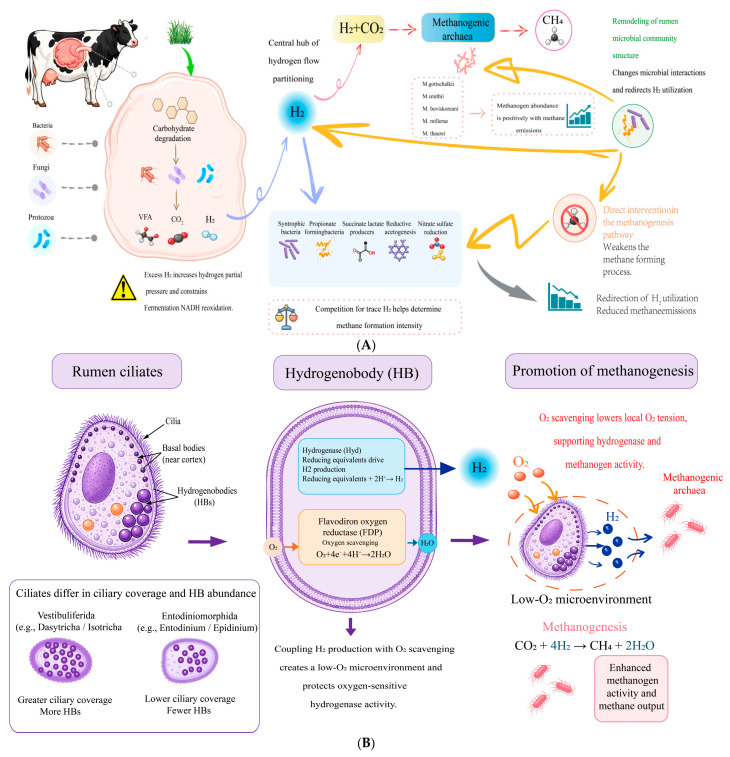
(**A**) Mechanisms and Strategies by Which Nutritional Regulation Reduces Methane Emissions in Ruminants. (**B**) Proposed mechanism by which rumen ciliates promote methanogenesis in ruminants. Rumen ciliates contain hydrogenobody (HB) organelles enriched in hydrogenase and flavodiiron oxygen reductase. Hydrogenase promotes H_2_ production, whereas flavodiiron oxygen reductase contributes to O_2_ scavenging, creating a low-oxygen microenvironment that supports hydrogenase activity and the activity of methanogenic archaea. Differences in ciliary coverage and HB abundance among ciliate lineages, including Vestibuliferida and Entodiniomorphida, may partly explain their lineage-specific methane-promoting potential. Adapted and redrawn based on recent mechanistic evidence.

Rumen fermentation is fundamentally governed by metabolic hydrogen flows, with CH_4_ serving as one of the major H_2_ sinks. This is consistent with the findings of Cord-Ruwisch et al. [24], who demonstrated that the competitive efficiency of different H_2_-consuming pathways for trace H_2_ depends on the thermodynamic advantage of their terminal electron acceptors. Consequently, whether H_2_ can be redirected to pathways other than methanogenesis is a critical factor determining the intensity of methane formation. Both fermentation product profiles and methane production are determined by the allocation of H_2_ among different metabolic pathways [25]. Therefore, the key to reducing methane emissions through nutritional regulation lies in reshaping the rumen microbial community, redirecting H_2_ utilization, and weakening methanogenic pathways (Figure 1A). Accordingly, nutritional strategies for enteric methane mitigation can be broadly organized into two interrelated categories: reshaping the rumen microbial community structure and directly intervening in methanogenic pathways.

### 2.1. Reshaping of the Rumen Microbial Community Structure

#### 2.1.1. Diet-Induced Alterations in the Rumen Microbial Community and Their Associations with Fiber Degradation Kinetics and Methane Production

Microbial community remodeling primarily occurs through the enrichment of alternative H_2_-utilizing bacterial populations, which suppress methanogenic archaea and their syntrophic networks, thereby reducing the flux of H_2_ into the methanogenesis pathway [12,18]. The rumen microbial community is not a static entity; rather, it undergoes continuous remodeling in response to host developmental stages and dietary transitions [12,19]. In a goat model, Wu et al. [12] observed that the rumen microbial community exhibited stage-specific co-oscillation with host transcriptional profiles, with Prevotella, Succinivibrio, Ruminococcus, and Fibrobacter becoming dominant during later developmental stages.

Within this dynamic remodeling framework, diet emerges as the most important external factor shaping the direction of hydrogen metabolism. Different dietary regimes selectively favor distinct H_2_ metabolic pathways and microbial assemblages. Compared with starch-based diets, fiber-based diets are more likely to enrich fiber-degrading bacteria, methanogenic archaea, and hydrogen-utilizing acetogenic bacteria, whereas starch-based diets tend to favor starch-degrading bacteria, lactate-utilizing bacteria, and respiratory H_2_-utilizing bacteria. These shifts promote the diversion of H_2_ toward propionate formation and other alternative hydrogen-utilization pathways, ultimately contributing to reduced methane emissions [19]. However, increasing the dietary concentrate proportion is commonly accompanied by decreased acetate concentration, increased propionate concentration, a reduced acetate-to-propionate ratio, lower ruminal *PH*, and impaired NDF digestibility [26]. Therefore, compared with simply increasing the dietary concentrate proportion, improving forage digestibility, optimizing roughage sources, and moderately adjusting the concentrate-to-roughage ratio while maintaining overall dietary balance may represent a more feasible nutritional strategy for mitigating methane emissions without compromising fiber utilization, rumen fermentation homeostasis, or animal performance.

Zhang et al. [4,25] demonstrated that different fiber sources can selectively shape distinct fiber-degrading microbial communities, thereby influencing roughage utilization efficiency. Bacteria and fungi are the principal contributors to fiber degradation in the rumen, providing up to 75% of the host’s energy supply through the enzymatic breakdown of plant cell walls into fermentable nutrients [27]. The effect of roughage on methane emissions largely depends on cell wall degradability [28]. Increased lignification, enhanced lignin–fiber cross-linking, and thickening of the secondary cell wall reduce NDF degradation rate and overall digestibility, thereby altering substrate release patterns and fermentation kinetics [28]. Following the fermentation of structural carbohydrates by fibrolytic microorganisms, metabolic flux is preferentially directed toward acetate and butyrate production, accompanied by increased H_2_ release [7,19]. If this H_2_ is not rapidly consumed by alternative electron-accepting pathways, it can be readily utilized as a substrate by hydrogenotrophic methanogenic archaea for CH_4_ production [7,19,28].

When the proportion of high-quality roughage in the diet increases, less lignified cell wall structures and an appropriate hemicellulose-to-cellulose ratio may facilitate rapid colonization and hydrolysis by fibrolytic bacteria, thereby shortening fermentation duration. This process may improve the temporal synchrony between hydrogen production and hydrogen utilization, reducing the accumulation of dissolved H_2_ in the rumen liquid phase and its subsequent utilization by methanogenic archaea. Concurrently, the fermentation profile may shift toward greater propionate production. Propionate-forming pathways, including the acrylate pathway and the succinate decarboxylation pathway, function as competitive hydrogen sinks by consuming reducing equivalents, thereby further redirecting hydrogen metabolic flux away from methanogenesis [7,25,28,29,30,31]. Several feed replacement studies have confirmed that increasing the proportion of high-quality roughage can reduce methane emissions while maintaining productive performance [30,31]. In lactating dairy cows, partial replacement of grass silage with corn silage reduced methane emissions per unit of dry matter intake or milk yield, accompanied by a decrease in the ruminal acetate-to-propionate ratio [28]. Similarly, studies in water buffalo have shown that high-quality roughages, such as alfalfa hay and whole-plant corn silage, improve dietary *NDF* and organic matter digestibility, whereas low-quality roughages, including rice straw and sugarcane tops, limit the accessibility of fibrolytic bacteria due to their higher lignin and silica contents and exhibit greater methane production potential [32].

#### 2.1.2. Functional Limits and Regulatory Strategies for Rumen Microbial Community Remodeling

Microbiome studies have further clarified the relationships among dietary composition, forage characteristics, and the functional structure of the rumen ecosystem. He et al. [32] reported that forage quality significantly affected the digestibility of dry matter (DM), organic matter (*OM*), and neutral detergent fiber (NDF) in water buffalo, as well as the ruminal acetate-to-propionate ratio. Meanwhile, single-cell transcriptomic analysis by Jia et al. [33] revealed marked functional specialization and heterogeneity among rumen microorganisms involved in fiber degradation, carbohydrate metabolism, and propionate-related pathways. These findings indicate that rumen microbial remodeling is not merely a shift in community composition, but also reflects functional redistribution among microbial taxa and metabolic pathways.

The methanogenic community may also be restructured during this process. For example, the relative abundance of genera with relatively lower hydrogen-utilization efficiency, such as Methanosphaera, may increase, whereas the abundance of the dominant methanogen Methanobrevibacter and its symbiotic association with ciliated protozoa may decrease [34]. Within the rumen microbial consortium, bacteria generally contribute more substantially to fermentation than fungi and protozoa [35]_._ Lignocellulosic feed is degraded through the synergistic activities of bacteria, protozoa, and fungi, with bacteria and fungi accounting for approximately 80% of the degradative activity, whereas protozoa contribute about 20% [36]. The high efficiency of the rumen microbiome in degrading complex plant cell walls is largely attributable to the coordinated actions of diverse microorganisms and their carbohydrate-active enzymes (CAZymes), which mediate the synthesis, degradation, and modification of glycosidic bonds. Crystalline cellulose is typically degraded through synergistic mechanisms involving endo- and exo-acting CAZymes, which target internal and terminal glycosidic bonds of polysaccharides, respectively [37]. Anaerobic fungi provide CAZymes that complement those of bacteria, and their hyphae or rhizoids can penetrate plant cell walls, thereby contributing to the breakdown of recalcitrant fibers. Genomic analysis by Li et al. [38] showed that Diplodiniinae and Ophryoscolecinae encode numbers of CAZymes comparable to those found in intestinal fungi, and that approximately 72% and 82% of their degradative CAZymes, respectively, target plant cell walls, indicating a strong potential for plant cell wall degradation in these two groups of rumen ciliates.

The role of protozoa in methane mitigation remains controversial. Three major viewpoints have been proposed. The first supports complete defaunation to improve nitrogen utilization efficiency and reduce methane emissions. The second argues against complete protozoal removal, emphasizing the important roles of protozoa in fiber degradation and rumen ecological stability [39]. The third advocates selective regulation of protozoal abundance or function rather than complete elimination, with the aim of balancing methane mitigation with rumen fermentation homeostasis [40]. However, the rumen is an integrated microbial ecosystem, and the broader consequences of protozoal regulation for microbial functions, nutrient metabolism, and host performance remain incompletely resolved. Recent mechanistic evidence has further refined our understanding of how rumen ciliates contribute to methanogenesis. Rumen ciliates have long been associated with enhanced methane production, but the cellular basis of this relationship has only recently been clarified. A recent study demonstrated that rumen ciliates harbor a hydrogen-producing organelle, termed the hydrogenobody (HB), which is enriched in hydrogenase and flavodiiron oxygen reductase. Through the coordinated production of H_2_ and scavenging of O_2_, this organelle may create a low-O_2_ microenvironment that supports oxygen-sensitive hydrogenase activity and favors methanogenic archaea. Importantly, the methane-promoting potential of rumen ciliates appears to differ among lineages. Vestibuliferida, such as Dasytricha and Isotricha, generally exhibit greater ciliary coverage and higher HB abundance than Entodiniomorphida, such as Entodinium and Epidinium, which may partly explain their stronger association with methanogenesis. These findings indicate that protozoa-mediated methane production should not be interpreted solely according to total protozoal abundance, but should also be considered in relation to ciliate lineage, HB abundance, interspecies H_2_ transfer, and O_2_-scavenging capacity (Figure 1B) [41].

#### 2.1.3. Species-Specific Responses to Nutritional Regulation Strategies and Their Application Boundaries

The effectiveness of dietary strategies and feed additives for methane mitigation varies markedly among ruminant species (Table 1 and Table 2). The numerical values presented in these tables should be interpreted as source-derived approximate responses that indicate the direction and relative magnitude of effects across species and intervention types, rather than as newly recalculated pooled estimates. When the same nutritional intervention is applied to different animals, its response may be modified by feeding behavior, rumen volume, digesta retention time, production stage, and host physiological status. These factors help explain why findings obtained from dairy cattle cannot be directly extrapolated to all ruminants. Although enteric methane emissions represent both an environmental burden and a loss of dietary energy at the production-system level, methanogenesis also functions within the rumen as an important hydrogen sink that helps maintain anaerobic fermentation and redox balance. Effective methane mitigation should therefore not rely solely on suppressing methane formation, but should aim to redirect hydrogen flux toward alternative metabolic pathways while maintaining rumen fermentation stability, fiber utilization, and animal performance. In this sense, methane mitigation should be understood not simply as “suppressing methane production,” but as reorganizing rumen metabolism toward greater efficiency. Beyond nutritional regulation, genetic background should also be considered when evaluating methane mitigation potential. Methane-related traits in ruminants show low-to-moderate to moderate heritability, with many estimates falling within approximately h^2^ = 0.2–0.4 depending on the trait definition, measurement method, breed, and production system [42]. This indicates that low-emission phenotypes can be transmitted across generations and that selective breeding may provide a cumulative and relatively stable mitigation pathway [43]. Compared with short-term dietary interventions, genetic improvement is slower but more permanent and additive over generations. Therefore, breeding for lower methane emissions should be regarded as a complementary strategy rather than a substitute for nutritional mitigation. Its practical application requires large-scale methane phenotype recording, reliable estimated breeding values, and careful consideration of genetic correlations with feed efficiency, productivity, fertility, and animal health [42]. Over the long term, the integration of low-methane breeding with nutritional regulation may contribute to population-level methane mitigation while maintaining production performance [42,43].

Optimizing the proportion and quality of forage is not only a fundamental approach for improving fiber degradation efficiency but also an important pathway for achieving synergistic improvements in production performance and environmental outcomes. The underlying mechanisms involve multiple levels, including fiber degradation kinetics, hydrogen metabolism redistribution, microbial community remodeling, and shifts in fermentation end-products. These changes may provide a more favorable ruminal environment for the subsequent use of methane-mitigation additives, such as lipids, nitrates, and 3-nitrooxypropanol.

It should be noted that methane mitigation and improved fiber utilization are not always aligned (Table 3). This trade-off can be understood from the role of methanogenesis in rumen redox balance and fiber fermentation. Methanogenesis represents an energy loss and an environmental burden, but it also serves as an important H_2_ sink that helps maintain a low ruminal H_2_ partial pressure and supports the reoxidation of reducing equivalents during anaerobic fermentation [23,25]. When methanogenesis is suppressed, the mitigation effect is more likely to be nutritionally favorable if H_2_ is redirected toward alternative sinks, such as propionate formation, reductive acetogenesis, or nitrate reduction. If such alternative pathways are insufficient, the accumulation of reducing equivalents may disturb fermentation balance rather than simply improve energy efficiency. Dietary strategies that reduce methane by increasing concentrate supply may also shift fermentation toward propionate and lower the acetate-to-propionate ratio, but excessive reductions in ruminal pH can inhibit cellulolytic bacteria and reduce NDF degradation [26]. Protozoal regulation provides another example of this complexity. Protozoa are associated with methanogens and can contribute to interspecies H_2_ transfer, yet they also participate in fiber degradation and help maintain rumen ecological interactions [39]. Therefore, methane mitigation should be evaluated together with fiber digestibility, dry matter intake, rumen fermentation stability, and animal performance, rather than being judged only by the magnitude of CH_4_ reduction.

The corn silage harvest maturity data summarized in Table 3 illustrate this trade-off under a specific dietary condition. These results are also consistent with broader evidence showing that silage characteristics, including starch content, NDF content, digestibility, and fermentation quality, can influence enteric methane yield in ruminants [50]. Increasing the harvest maturity of whole-plant corn silage reduced the degradation rates and digestibility of starch and NDF, while methane yield and methane intensity also decreased. This pattern suggests that a reduction in methane emissions does not necessarily indicate improved fiber utilization. The practical objective should therefore not be to achieve the lowest possible methane output, but to identify a balance among methane emissions, fiber digestibility, rumen homeostasis, and productive performance under specific production conditions [28].

#### 2.1.4. Regulatory Effects of Plant-Derived Secondary Metabolites

Plant-derived secondary metabolites, particularly condensed tannins and saponins, regulate methane emissions in ruminants mainly by modifying rumen microbial structure and fermentation patterns, rather than by directly targeting methanogenic archaea [51,52].

Methanogenic archaea, mainly members of the genus Methanobrevibacter, that are attached to the surface of protozoa or occur intracellularly can contribute approximately 9–25% of total ruminal methane production [15]. Saponins may inhibit protozoal growth by altering cell membrane permeability, thereby weakening protozoa–methanogen symbiosis, reducing the directed transfer of hydrogen from protozoa to methanogens, and shifting fermentation end-products toward propionate [15,53]. The bioactivity of saponins from different plant sources, such as tea saponins, yucca saponins, and alfalfa saponins, varies considerably, and their effects are influenced by the dietary forage-to-concentrate ratio and supplementation level [54,55].

Unlike saponins, condensed tannins mainly regulate methane production through three mechanisms. First, they form pH-reversible complexes with dietary proteins, thereby reducing rapid ruminal protein degradation and ammonia release, which may decrease hydrogen production associated with protein fermentation [56]. Second, certain tannin components may directly inhibit the adhesion and growth of methanogenic archaea [57]. Third, tannins can exert dose-dependent effects on fiber-degrading bacteria, such as Ruminococcus flavefaciens: low concentrations may promote fiber attachment, whereas high concentrations may inhibit fiber-degrading enzyme activity [58]. Within an appropriate inclusion range, generally 2–4% of dietary dry matter, condensed tannins may reduce methane emissions while maintaining feed intake and fiber digestibility. However, excessive supplementation may impair digestion by inhibiting fiber-degrading bacteria or by forming poorly degradable tannin–fiber complexes [53].

Under production conditions, low to moderate levels of tannins or saponins generally do not adversely affect milk yield or growth performance, and some studies have reported improved nitrogen utilization efficiency and reduced urinary nitrogen excretion [59,60]. Saponins mainly reduce ammonia production and influence microbial protein synthesis by suppressing protozoa and modulating the rumen microbiome. In contrast, tannins improve nitrogen utilization primarily by forming complexes with dietary proteins, reducing rapid ruminal protein degradation, and shifting nitrogen excretion from urine to feces [35,37]. By binding to proteins, tannins can decrease the ruminally degradable protein fraction and increase the proportion of undegraded dietary protein, thereby enhancing the flow of absorbable amino acids to the small intestine. Meanwhile, tannins may reduce ruminal ammonia concentration and blood urea nitrogen levels, thus decreasing the proportion of nitrogen excreted as urinary urea. This nitrogen-shifting effect is more pronounced in grazing systems or diets with a high proportion of forage [53].

Tannins and saponins show mechanistic complementarity in nitrogen metabolism and production performance rather than a fully confirmed synergistic effect. At appropriate inclusion levels, neither component generally impairs production performance, and both may improve nitrogen use efficiency. However, the potential synergistic effects of their combined use still require further confirmation through in vivo studies [15,53,60,61,62,63]. Nevertheless, the methane-mitigation effects of tannins and saponins should be interpreted with caution, as substantial heterogeneity has been reported among published studies. This variability is likely related to differences in plant source, chemical structure, tannin type, molecular weight, degree of polymerization, saponin composition, supplementation dose, basal diet composition, forage-to-concentrate ratio, animal species, physiological stage, and experimental duration. In the case of tannins, low to moderate inclusion levels may reduce methane emissions through changes in rumen fermentation, weakened methanogen-associated microbial interactions, or improved nitrogen partitioning. Once the inclusion level becomes excessive, however, tannins may depress dry matter intake, inhibit cellulolytic bacteria, reduce fiber degradation, and form poorly degradable tannin–protein or tannin–fiber complexes. Under these conditions, the observed decline in methane emissions may partly result from reduced intake or impaired fermentation rather than from a genuine improvement in rumen efficiency. Responses to saponins are similarly context dependent. Their antiprotozoal activity may weaken protozoa–methanogen associations and thereby reduce methane formation, but excessive suppression of protozoa may disturb rumen microbial balance and compromise fiber digestion. Therefore, plant-derived secondary metabolites are more likely to be effective when applied at moderate doses under compatible dietary conditions and when rumen fermentation, feed intake, and fiber utilization are maintained. By contrast, their efficacy is likely to be limited, or even counterproductive, when high inclusion levels impair digestion, reduce intake, or are applied without adequate consideration of animal species, physiological state, and basal diet characteristics.

Overall, plant-derived secondary metabolites should be regarded as context-dependent supplementary tools rather than universally effective methane inhibitors. Their emission-mitigation effects need to be validated under specific dietary backgrounds, controlled dosage conditions, and appropriate animal production stages. In grazing or low-input systems dominated by forage-based diets, locally available tannin- or saponin-rich plant resources may offer both economic feasibility and environmental adaptability [16]. Future studies should integrate metagenomics and metabolomics to elucidate the selective inhibitory effects of different plant-derived secondary metabolites on specific methanogenic archaeal groups, such as Methanobrevibacter ruminantium and Methanosphaera stadtmanae, and long-term feeding trials are needed to verify their stability and economic feasibility throughout the production cycle.

### 2.2. Direct Intervention in Methane Production Pathways

Compared to community restructuring, interventions targeting methane production pathways place greater emphasis on directly blocking the terminal metabolic steps of methanogenic archaea, thereby triggering secondary reorganization of the ruminal H_2_ metabolic network [21,64]. Methane emissions from ruminants represent a net loss of feed energy, typically accounting for approximately 2–12% of total feed energy [6]. The wide range of these fluctuations also indicates their potential for intervention. This is not merely an environmental issue but also a matter of inefficient energy utilization. Based on this understanding, researchers have begun to seek strategies that can directly intervene in methane production pathways without significantly impairing feed intake or production performance; among these, *3-NOP* is one of the most extensively studied representative inhibitors [17]. Hristov et al. [65] found that adding 40–80 mg/kg DM of 3-NOP for 12 consecutive weeks in high-yielding lactating dairy cows resulted in a steady reduction of approximately 30% in gastrointestinal methane emissions.

At the same time, there were no significant negative effects on dry matter intake, milk yield, or feed efficiency. Recent meta-analytic evidence further supports the effectiveness of 3-NOP in reducing enteric methane emissions, while also indicating that the magnitude of response may vary with animal type, additive dose, diet composition, and production context [47]. *3-NOP* specifically targets methyl-coenzyme M reductase (*MCR Methyl-coenzyme M reductase*), a key enzyme in the final step of methane production. Molecular docking, purified enzyme activity assays, and EPR results further indicate that *3-NOP* inactivates *MCR* by oxidizing the *Ni*(*I*) at its active site, thereby inhibiting methane production in methanogenic archaea [66]. Inhibition of *MCR* leads to decreased expression of related genes such as mcrA, as well as reduced abundance of dominant methanogenic archaea like Methanobrevibacter. Concurrently, certain regulatory mechanisms promote the proliferation of bacterial communities such as Prevotella, thereby influencing hydrogen utilization and reducing methane production [21,64].

Metagenomic and metatranscriptomic studies indicate that the inhibitory effect of 3-NOP varies across different lineages of methanogenic archaea. Its inhibition of Methanobrevibacter is generally stronger than that of Methanosphaera, and this response is accompanied by a downward trend in MCR-related transcripts. Following the suppression of methane production, ruminal H_2_ metabolism does not simply reflect the general enhancement of a single alternative hydrogen sink, but is accompanied by the upregulation of hydrogenase expression and enzymes related to butyrate synthesis. Recent mechanistic evidence further indicates that inhibition of hydrogenotrophic methanogenesis can remodel ruminal microbial fermentation and stimulate alternative hydrogen-utilizing pathways, such as acetogenesis, supporting the view that methane mitigation involves hydrogen flux redistribution rather than only suppression of the terminal methanogenic step [5]. The inhibition of these pathways triggers a readjustment of the methanogenic lineage, H_2_ production/sensing systems, *VFA* composition, and even protozoa and certain nitrogen metabolism pathways [67]. Comparative metagenomic analysis further indicates that the microbial responses induced by *3-NOP* are not entirely consistent between beef and dairy cattle systems: overall, they are characterized by suppression of *mcrA* and hydrogen-dependent methanogenesis-related pathways, decreased abundance of Methanobrevibacter gottschalkii and the protozoan Isotricha prostoma, and increased abundance of Methanosphaera, certain Prevotella species, and metabolic pathways associated with propionate and butyrate production [21]. Recent meta-analyses further show that the efficacy of 3-NOP is influenced by animal type, diet composition, additive dose, and supplementation duration. Therefore, *3-NOP*-mediated intervention in methane production pathways is not merely the inhibition of a single terminal reaction, but rather a systemic process accompanied by the synergistic remodeling of methanogenic communities, hydrogen metabolism networks, and related fermentation pathways. Its emission-reduction effects and metabolic responses are jointly influenced by factors such as host type, diet composition, additive dosage, and the duration of intervention.

## 3. Key Nutritional Strategies for Reducing Nutrient Excretion in Ruminants

**Figure 2:**
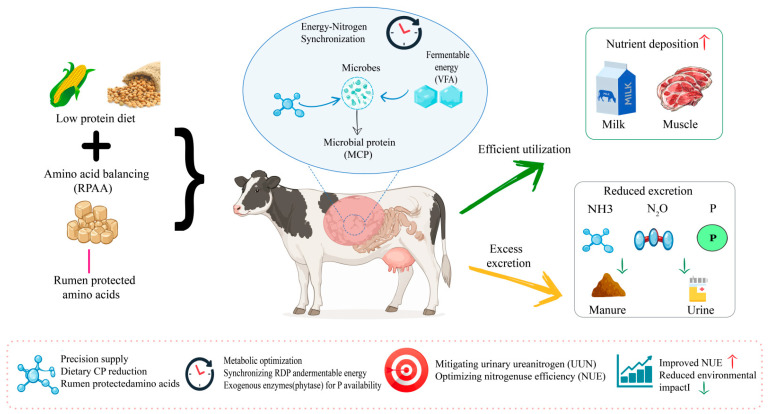
Nutritional strategies for reducing nitrogen and phosphorus excretion in ruminants.

The key to reducing nutrient excretion through nutritional regulation is to improve the efficiency of nutrient digestion, absorption, and retention in the animal body, thereby reducing the amount of nitrogen and phosphorus entering feces and urine at the source, rather than simply lowering dietary nutrient supply. NH_3_ and N_2_O emissions from livestock manure account for 49% and 30% of total agricultural emissions, respectively, and more than 15% of nitrogen intake in cattle is excreted in manure [68]. Unlike methane mitigation, which mainly focuses on the regulation of rumen fermentation, control of nutrient excretion emphasizes source reduction through precision feeding and optimization of nutrient metabolic pathways (Figure 2) [69,70].

### 3.1. Regulating Nitrogen Excretion: From Crude Protein Reduction to Microbial Protein Synthesis

Ruminants do not have a direct requirement for crude protein per se; rather, their actual requirement is for absorbable amino acids, which are mainly derived from rumen-undegradable protein and rumen microbial protein [71]. Therefore, reducing nitrogen excretion depends largely on improving the efficiency with which dietary nitrogen is converted into microbial protein and retained in host tissues. Apelo et al. [72] found that, when metabolizable protein requirements were met, reducing dietary crude protein from 17% to 15% in lactating dairy cows did not significantly affect feed intake or milk production, while milk urea nitrogen concentrations decreased in all low-protein treatments. However, the effects of low-protein diets supplemented with rumen-protected amino acids remain inconsistent. Van den Bossche et al. [73] reported that supplementation with rumen-protected amino acids in a diet containing 14.5% crude protein did not further improve nitrogen efficiency, suggesting that the mitigation effect of low-protein diets depends on a balanced supply of absorbable amino acids.

In addition to crude protein level, the degradation characteristics of protein fractions also influence nitrogen utilization pathways. Zhang et al. [11] found in sheep that reducing dietary crude protein while maintaining the proportion of soluble protein at approximately 25–30% decreased plasma urea nitrogen, upregulated the expression of small intestinal amino acid transporters, and reduced fecal reactive nitrogen emissions. These findings indicate that optimizing protein composition can improve intestinal nitrogen absorption and microbial nitrogen transformation in a coordinated manner.

Thus, reducing crude protein levels, maintaining a balanced supply of limiting amino acids, and optimizing protein fraction characteristics are key nutritional strategies for reducing nitrogen excretion in ruminants. The focus should not be placed simply on lowering protein input, but rather on improving the overall efficiency of nitrogen utilization in the rumen and intestine, so that more nitrogen is directed toward microbial protein synthesis and host nutrient retention rather than being excreted as urea or reactive nitrogen.

The efficiency of rumen microbial protein synthesis is a central determinant of nitrogen partitioning [74,75]. When the rate of ammonia release exceeds the capacity of microbial assimilation, excess ammonia is converted to urea and excreted via the urea cycle. Therefore, synchronizing the supply of energy and nitrogen is essential for enhancing microbial protein synthesis. In recent years, regulation of ruminal urease activity has attracted increasing attention. Urea is rapidly hydrolyzed by urease in the rumen to produce ammonia, whereas microbial assimilation often lags behind, resulting in ammonia accumulation and increased urinary nitrogen excretion [76,77,78]. Accordingly, moderate inhibition of urease activity may slow ammonia release and promote the incorporation of urea nitrogen into microbial protein.

He et al. [76] demonstrated, through molecular docking and in vitro rumen fermentation assays, that coptisine strongly inhibits rumen bacterial urease, thereby significantly reducing NH_3_ release and delaying urea degradation. More recent studies have further shown that epiberberine targets the urease accessory protein UreG, inhibits its GTPase activity, weakens nickel–UreG binding, and alters protein conformation, ultimately leading to lower ammonia release and slower urea degradation in an in vitro rumen microbial fermentation system [79]. Subsequent studies have also indicated that urease inhibition is not limited to reducing peak ammonia release, but may also alter the partitioning of urea nitrogen within the rumen. Zhang et al. [68] further confirmed that allicin promotes the incorporation of urea nitrogen into microbial amino acids and nucleotides by inhibiting urease activity and reshaping the rumen microbial community. However, the practical efficacy of urease inhibitors is influenced by the dietary context, dosage, and production stage, and their long-term stability and economic feasibility require further validation in vivo.

To improve the clarity of this section, representative studies on dietary crude protein reduction, amino acid balance, protein fraction regulation, and ruminal urease inhibition are summarized in Table 4. These studies indicate that nitrogen-excretion mitigation should not be understood simply as lowering dietary crude protein input, but rather as improving nitrogen capture and partitioning through coordinated regulation of absorbable amino acid supply, ruminal ammonia release, microbial protein synthesis, and host nitrogen retention.

### 3.2. Regulation of Phosphorus Excretion: From Precision Phosphorus Supply to Phytate Phosphorus Utilization

Phosphorus is an essential element involved in energy metabolism, nucleic acid synthesis, and skeletal mineralization in animals [80,81]. However, when dietary phosphorus supply exceeds animal requirements, approximately 87% of the ingested phosphorus may be excreted via feces [82,83]. Moreover, an increase in the proportion of water-soluble phosphorus can further aggravate the risk of environmental phosphorus loss [84]. Excessive phosphorus supplementation remains common in intensive production systems. A survey by Dou et al. [85] showed that the average dietary phosphorus concentration in lactating dairy cows was 34% higher than the NRC recommendation, with the excess ranging from 6% to 64% during mid- and late lactation [86]. Therefore, the primary strategy for reducing phosphorus excretion is to implement precision phosphorus feeding based on updated nutrient requirement models, such as *NASEM 2021*, and to minimize unnecessary “safety margins” [87]. In addition to available phosphorus supply and phytase supplementation, the dietary calcium-to-phosphorus ratio is also an important factor influencing phosphorus absorption, utilization, and excretion [88]. Calcium and phosphorus metabolism are closely linked through intestinal absorption, skeletal mineralization, salivary recycling, and endogenous fecal losses [77]. An imbalanced Ca:P ratio may alter mineral availability and affect the efficiency with which dietary phosphorus is retained or excreted. Therefore, phosphorus mitigation should not rely only on reducing total phosphorus input or improving phytate phosphorus utilization; it should also consider the coordinated supply of calcium and phosphorus according to animal requirements, production stage, dietary composition, and mineral source [77,88]. In practical formulation, maintaining an appropriate Ca:P balance can help support mineral homeostasis while avoiding excessive phosphorus supplementation and unnecessary fecal phosphorus losses.

Under low-phosphorus dietary conditions, the application value of exogenous phytase needs to be reconsidered. Although rumen microorganisms in ruminants possess some capacity to degrade phytate, this degradation is limited in efficiency. Winter et al. [89] reported that phytase supplementation in low-phosphorus diets did not significantly improve total-tract phosphorus digestibility. In contrast, Giagnoni et al. [90] found that early-lactation dairy cows responded more positively to phytase supplementation than mid-lactation cows, and that excessive dietary phosphorus levels may mask the effects of phytase. Furthermore, Dersjant-Li et al. [91] demonstrated that, when total phosphorus intake was strictly controlled, exogenous phytase increased phytate phosphorus digestibility and reduced fecal phosphorus excretion in a dose-dependent manner. Therefore, exogenous phytase is more appropriately regarded as a complementary strategy following precision phosphorus feeding, rather than a universal substitute for basic phosphorus management.

### 3.3. Interface Regulation and Region-Specific Adaptation Strategies

Nutrient-loss mitigation in ruminant production should be understood as a coordinated process that begins with dietary source control and is then complemented by interface regulation during manure handling, land application, and runoff transport. Precision protein and phosphorus feeding can reduce the amount of nitrogen and phosphorus entering manure at the source, whereas interface regulation aims to limit the subsequent transfer of these nutrients from manure or soil surfaces to the surrounding environment [69,70,92,93,94,95]. In this sense, interface regulation should not be regarded as a substitute for nutritional regulation, but rather as a complementary component of nutrient-loss mitigation, especially when combined with feasible dietary source control. It should also be acknowledged that, in some farms or regions where routine feed analysis, precise diet formulation, stable feed supply, or technical support is limited, manure treatment and soil-interface management may represent essential and immediately applicable mitigation measures.

This principle is particularly important for phosphorus management. When dietary phosphorus supply exceeds animal requirements, unnecessary fecal phosphorus excretion increases the environmental pressure on manure handling and land application systems [69,82,83,84,85]. Under such conditions, the priority should remain precision phosphorus feeding, including updated requirement-based formulation, avoidance of excessive safety margins, and consideration of dietary calcium-to-phosphorus balance [69,87,88]. Once phosphorus input has been controlled at the dietary level, manure- and soil-interface measures can further reduce phosphorus mobilization and transport. For example, chemical amendment of dairy cattle slurry with hydroxylated polyaluminum chloride or alum can reduce dissolved and particulate phosphorus losses in runoff [92]. Polyacrylamide can also decrease nutrient transport by improving soil aggregate stability and reducing sediment-associated nutrient losses [93,94,95]. These approaches can provide important protection against phosphorus mobilization and runoff losses, particularly in systems where precision feeding is difficult to fully implement. However, when dietary phosphorus input remains excessive, interface regulation may face greater pressure and is likely to be more effective when combined with reasonable dietary phosphorus management [69,92,93,94,95].

A similar logic applies to nitrogen mitigation. Low-protein diets with balanced amino acid supply, improved rumen microbial protein synthesis, and appropriate regulation of ruminal ammonia release can reduce urinary nitrogen excretion at the source [68,70,72,73,74,75,76,77,78,79]. Nevertheless, the environmental outcome also depends on manure handling, land application conditions, soil properties, and the potential for nitrogen transformation into ammonia or nitrous oxide [69,70,92,93,94,95]. Practical mitigation should therefore combine dietary nitrogen-use efficiency with manure management strategies that reduce nitrogen volatilization, leaching, and runoff risks. Nutritional regulation and interface regulation are thus better viewed as linked components of the same nutrient-flow chain rather than as independent interventions.

The regional suitability of these strategies depends strongly on feed resources, production intensity, climate conditions, management capacity, and economic feasibility (Table 5 and Table 6) [44,52,96]. In temperate intensive systems, where total mixed ration feeding and high-quality silage are commonly available, precision protein–energy formulation, precision phosphorus feeding, and feed additives with relatively consistent responses may be more feasible [44,69,87]. In tropical and subtropical systems, the higher lignification of forage resources, seasonal feed fluctuations, and heat stress may limit the stability of some high-cost inhibitors; strategies based on forage improvement, agricultural byproduct pretreatment, and locally available plant secondary metabolites may therefore be more practical [44,52,96]. In resource-limited grazing or semi-grazing systems, low-cost forage improvement and locally available tannin- or saponin-rich plant resources may be more suitable than supply-chain-dependent additives [52,96]. For grassland–supplementary feeding mixed systems, fixed year-round use of a single mitigation technology is unlikely to be appropriate, because dietary background and animal requirements differ substantially between grazing and supplementation periods [44,96].

Taken together, the practical selection of mitigation strategies should begin with reducing avoidable nutrient excess through precision feeding, while maintaining rumen fermentation stability and animal performance. Where nutrient transfer risks remain high, manure- or soil-interface measures can provide additional protection against environmental losses. The final choice of strategy should then be adjusted according to regional feed resources, economic cost, labor capacity, and long-term feasibility [44,69,87,92,93,94,95,96]. This framework may help link the mechanisms discussed in the previous sections with practical management decisions under different production conditions.

## 4. Methodological Innovations and Future Perspectives

The evaluation of nutritional regulation strategies relies on a multi-scale methodological framework that extends from in vitro screening and in vivo validation to system-level environmental assessment. Although considerable methodological progress has been made, gaps remain in the integration of evidence across different scales. In vitro methane detection systems, environmental metabolomics, LCA (life cycle assessment), and EC (eddy covariance) techniques do not directly reduce emissions; however, they provide essential support for the rapid identification of nutritional strategies, mechanistic interpretation of their effects, and evaluation of environmental benefits within broader production systems.

This need for cross-scale methodological integration is particularly important for methane research, because methane emissions must be measured and interpreted across different biological, animal, facility, and ecosystem scales. Existing methods for methane quantification cover multiple levels, including individual animals, housing facilities, and large-scale environmental systems. No single technique is universally superior across all scenarios; therefore, method selection should be guided by the specific research objective [97]. Similarly, in vitro methane detection is not a single method but a group of technical systems, including pure-culture systems, batch culture systems, continuous culture systems, and their derivatives. These methods differ substantially in their applicable scenarios, information output, and extrapolation boundaries.

Table 7 does not represent a single in vitro methane detection method, but rather a set of technical systems designed for different levels of research. Pure-culture systems are more suitable for narrowing the research question to specific microbial strains or metabolic pathways, thereby helping to identify why a particular target responds to an intervention. Batch culture systems are more appropriate for early-stage screening, as they allow rapid preliminary comparisons among multiple candidate feed ingredients, additives, or dosage combinations under standardized culture conditions. Continuous culture systems and their derivatives further shift the research focus toward fermentation stability, making them more suitable for observing whether methane production, substrate degradation, and microbial adaptation persist over longer culture periods. Therefore, the value of in vitro methane detection systems lies not in replacing in vivo experiments, but in providing stratified methodological support for specific research questions and enabling different techniques to be applied at the most appropriate stages.

Among these methods, batch culture systems are widely used in ruminant methane research because they are relatively simple to operate and allow multiple treatments to be compared within a short period. By simultaneously measuring total gas production, methane production, volatile fatty acids, *pH*, and NH_3_-N, researchers can rapidly assess whether a given treatment merits further in vivo validation. This advantage, however, is accompanied by clear limitations. Batch culture is essentially a closed system, in which the continuous accumulation of fermentation end products and gases may inhibit fermentation. Its results are therefore more suitable for directional judgment and preliminary screening than for direct extrapolation to emission levels under real feeding conditions [98]. The predictive value of in vitro methane screening should be assessed by its ability to identify candidate treatments for subsequent animal trials, rather than by its capacity to directly estimate absolute in vivo methane emissions [98,100]. Evidence from validation studies suggests that in vitro systems can capture the direction of some animal-level responses, although their quantitative agreement with in vivo outcomes remains variable. Hatew et al. [104] reported that 24-h in vitro CH_4_ production was associated with in vivo CH_4_ production when expressed per unit of rumen-fermentable organic matter, whereas this relationship was not observed when CH_4_ production was expressed per unit of organic matter intake. This finding indicates that the choice of response metric can strongly influence the apparent agreement between in vitro and in vivo measurements. In a study using plant-derived compounds, Martínez-Fernández et al. [105] found that propyl propane thiosulfinate and bromochloromethane produced larger methane reductions in vitro than in vivo, suggesting that in vitro systems may overestimate the mitigation magnitude of some bioactive compounds. Taken together, these studies indicate that in vitro screening is useful for ranking candidate strategies and identifying promising mechanisms, but it should not be used as the sole basis for predicting animal-level mitigation efficacy [98,100,104,105].

For practical screening, candidate treatments may be advanced to in vivo validation when they produce a consistent and biologically meaningful reduction in methane production in vitro, for example by approximately 20% or more, while maintaining total gas production, VFA concentration, pH stability, ammonia-N concentration, and fiber degradation within acceptable ranges. This threshold should be interpreted as a preliminary decision rule rather than a universal standard. A large apparent reduction in methane should not be considered favorable if it mainly results from suppressed fermentation, reduced substrate degradation, or impaired microbial activity. Conversely, a moderate methane reduction may still be valuable when it is accompanied by stable fermentation, maintained fiber digestion, improved nitrogen utilization, or clear practical feasibility. For this reason, in vivo validation remains necessary before any strategy identified through in vitro screening can be recommended for practical feeding systems [98,100,104,105].

When the research objective extends beyond short-term screening and aims to evaluate fermentation stability and sustained responses, continuous culture systems and their derivatives become more important. *RUSITEC* was developed to meet this need. In the original design of this long-term rumen simulation technique, the objective was to maintain a rumen microbial community similar to that under normal ruminal conditions within a strictly controlled system, while also enabling quantitative gas collection and measurement. After reaching a steady state, the system can maintain fermentation characteristics similar to those of the donor animal’s rumen for an extended period, and normal protozoal populations may even be maintained for up to 49 days. This makes *RUSITEC* more suitable for bridging short-term screening and sustained-effect evaluation [99].

Nevertheless, although continuous culture systems are closer to continuous fermentation than batch culture systems, their limitations remain evident. In vitro platforms cannot fully reproduce the fluctuating and interrelated factors present in real animal feeding conditions, including feeding rhythm, rumen digesta flow, nutrient absorption, and host physiological regulation. For this reason, in vitro methane detection techniques are more appropriate as front-end identification tools. They can help researchers determine which strategies merit further investigation and can provide mechanistic clues, but they cannot independently determine whether a nutritional strategy will maintain stable methane mitigation under practical feeding conditions. Nor can they fully reveal whether methane reduction is accompanied by synchronous changes in other metabolic processes or environmental outcomes.

For many nutritional interventions, changes in methane production are relatively easy to observe. The more complex question is whether these changes arise from the redistribution of hydrogen flow or from alterations in methanogenesis-related metabolic pathways [19,21]. Moreover, the issue often extends beyond the rumen itself, because the reconfiguration of microbial functional networks is frequently accompanied by host responses. This is why multi-omics studies increasingly emphasize microbiome–host interactions [10,18]. At the same time, rumen microorganisms do not respond as a uniform entity; substantial heterogeneity exists among different functional groups [33]. In this context, the value of metabolomics is not simply that it adds several additional indicators, but that it enables methane emissions, substrate conversion, and nitrogen metabolism to be interpreted within a unified metabolic framework, thereby providing deeper evidence for the mechanistic interpretation of nutritional strategies [11].

Environmental metabolomics also has limitations. Environmental sample matrices are highly complex, and metabolite extraction, annotation, and quantification remain challenging. In addition, the comparability of results across analytical platforms is still limited. Therefore, environmental metabolomics is more suitable for integrated interpretation with metagenomic, transcriptomic, and other omics data than for use as the sole basis for mechanistic conclusions [106].

Mechanistic explanation does not necessarily confirm environmental effectiveness under real production conditions. Once research moves from culture systems and confined feeding trials to open grazing systems, the key question is no longer merely whether a treatment changes rumen fermentation, but how greenhouse gas fluxes change at the system scale when animals are continuously moving, meteorological conditions fluctuate, and emission sources are spatially variable. The value of eddy covariance technology lies precisely at this level. It is more suitable for continuous monitoring of net CO_2_ and *CH*_4_ exchanges at the herd, plot, or ecosystem scale, and therefore functions more as a validation tool under real-world conditions than as a preliminary screening platform [107].

Even so, changes in gas flux alone cannot be directly equated with environmental benefits. In natural grassland grazing systems, evaluations that focus only on enteric methane emissions or product-based emission intensity may underestimate the importance of soil organic carbon dynamics and the net carbon balance of the entire system. Previous studies have emphasized that assessment of natural grassland systems should not be limited to a single emission indicator, but should also incorporate ecosystem net exchange, changes in soil organic carbon, and complete carbon budgets [3]. LCA provides a useful tool for comprehensive assessment of environmental costs at the system scale, but its conclusions are highly dependent on the definition of system boundaries [108]. If plant carbon uptake and soil organic carbon changes are ignored, the results may favor high-input alternative strategies while underestimating the mitigation potential of well-managed grazing systems [3,109].

Therefore, ecosystem-scale fluxes, product outputs, and carbon budgets should be considered within a unified evaluation framework. EC can provide direct evidence of ecosystem-scale gas fluxes, while LCA can link these data with production processes and environmental costs under clearly defined system boundaries. Such integration may provide a more comprehensive assessment of the net environmental benefits of real production systems.

From this perspective, the future priority is not simply to accumulate more individual emission-reduction measures, but to establish an integrated framework capable of explaining these changes across scales. Nutritional inputs alter substrate structure and hydrogen release patterns; microbial community restructuring affects metabolic pathways and competitive relationships; and host feeding behavior, digestive efficiency, and production level further modify the direction and magnitude of these responses. At the environmental level, methane intensity, nitrogen and phosphorus losses, and system-level net carbon balance together determine the final outcome.

Future research should therefore move beyond asking whether a single strategy is effective. Instead, it should evaluate whether nutrition, microorganisms, host responses, and environmental outcomes can be integrated into the same explanatory framework, while also incorporating cost, adaptability, and net environmental benefits into practical decision-making. Such cross-scale integration may help determine the conditions under which green nutritional technologies can move from locally effective responses under controlled or laboratory conditions toward more stable and practically applicable management strategies at the farm scale.

## 5. Limitations and Future Directions

Although substantial progress has been made in nutritional strategies for mitigating emissions from ruminants, several limitations should be acknowledged. The interpretation of the current literature should consider the possibility of publication bias, as studies reporting clear mitigation effects may be more visible in the published literature than those showing limited or inconsistent responses. This may influence the overall assessment of the apparent efficacy and robustness of some nutritional strategies. Future systematic reviews and meta-analyses should therefore incorporate explicit assessments of publication bias, including funnel plots, sensitivity analyses, and, where possible, unpublished or grey literature.

The interpretation of existing findings is also constrained by the experimental conditions under which many nutritional interventions have been tested. A large proportion of studies have been conducted in respiration chambers, in vitro batch culture systems, or short-term feeding trials. Although these approaches are valuable for mechanistic investigation and initial screening, their results cannot be directly extrapolated to long-term commercial production systems. This issue is particularly relevant for 3-NOP supplementation, nitrate, plant secondary metabolites, and urease inhibitors, because their practical efficacy may vary with animal type, diet composition, production stage, additive dose, adaptation period, and farm management conditions.

Another limitation is that methane mitigation is often evaluated primarily according to the magnitude of enteric CH_4_ reduction, while broader nutritional and productive consequences receive less attention. Some strategies may alter dry matter intake, fiber digestibility, volatile fatty acid profiles, nitrogen utilization, rumen microbial stability, or animal performance. Thus, methane mitigation and improved nutrient utilization are not always fully aligned. Future studies should integrate methane emissions, nitrogen and phosphorus excretion, animal productivity, rumen microbial function, and host metabolic responses within the same analytical framework.

Economic feasibility represents a further challenge for the practical application of nutritional mitigation strategies. Additives such as 3-NOP have shown clear methane-mitigation potential, but their adoption in commercial production will depend on additive cost, delivery method, diet type, feeding system, farm management conditions, and the availability of carbon credits or other economic incentives. For this reason, future research should move beyond biological efficacy alone and incorporate cost-effectiveness, farmer adoption, and life cycle-based environmental benefits.

A more integrated system-level perspective is also needed. The environmental benefits of nutritional mitigation strategies may differ depending on system boundaries, functional units, allocation methods, and whether feed production, manure management, land-use change, and soil carbon dynamics are included. Combining animal-level measurements, long-term feeding trials, rumen multi-omics, economic analysis, and life cycle assessment will be essential for determining whether these strategies can provide stable, practical, and economically feasible mitigation benefits under real production conditions.

## 6. Conclusions

Achieving sustainability in ruminant production may require more coordinated regulation of the entire “rumen fermentation–microbial interaction–host metabolism–environmental emissions” chain through nutritional strategies. Methane mitigation largely depends on regulating ruminal hydrogen flow, methanogenic pathway activity, and microbial ecological networks, whereas the control of nitrogen and phosphorus excretion relies on precision nutrient supply, improved utilization efficiency, and the coordinated optimization of nutrient partitioning within and beyond the animal.

Strategies such as improving forage quality, adjusting the forage-to-concentrate ratio, applying plant-derived bioactive compounds and additives such as 3-nitrooxypropanol (3-NOP), adopting low-protein diets with balanced amino acid supply, implementing precision phosphorus feeding, and conditionally supplementing phytase can contribute to emission mitigation and nutrient loss reduction. However, the effectiveness of these measures should be comprehensively evaluated in terms of their combined effects on production performance, rumen homeostasis, resource utilization, and environmental benefits.

Future research should therefore move beyond evaluating the effectiveness of individual strategies in isolation. A more useful direction may be to develop cross-scale analytical frameworks that connect nutritional inputs with rumen microbial functions, host metabolic responses, production performance, and environmental outcomes. Mechanistic rumen fermentation models could help describe hydrogen flow, VFA production, and shifts in methanogenic pathways, whereas systems biology models may provide a basis for integrating microbial and host metabolic responses. Machine learning approaches may also be useful for combining multi-omics profiles with diet composition, animal performance, and emission data, especially when large and heterogeneous datasets become available. At the production-system level, LCA-linked decision models could further support the evaluation of environmental and economic trade-offs under different feeding and management conditions. Together, these approaches may help identify the dietary, microbial, host, and management contexts in which green nutritional technologies can move from locally effective responses under controlled or laboratory conditions toward more stable and practically applicable strategies at the farm scale.

## Figures and Tables

**Table 1:** Comparison of the methane mitigation effects of different dietary strategies across ruminant species.

Strategy	Dairy Cattle	Beef Cattle	Sheep	Evidence Notes	References
Improved forage quality	CH_4_ production +8%; CH_4_ yield −10%; CH_4_ intensity −19%	CH_4_ production +7%; CH_4_ yield ≈ 0	CH_4_ yield as %GEI +7%	Responses are most consistent in dairy cattle.	[44,45]
Corn silage replacement	DMI +11%; CH_4_ yield −5%; CH_4_ intensity −8%	DMI −5%; CH_4_ yield ≈ 0	Limited or nonlinear response	Effects are clearer in dairy cattle.	
Legume inclusion	DMI +16%; CH_4_ yield −17% to −18%	Limited evidence	DMI −5%; CH_4_ yield +2%	Evidence is limited or inconsistent outside dairy cattle.	
Tannin-rich forages	DMI +20%; CH_4_ yield −16%; CH_4_ intensity −18%	Limited evidence	DMI +34%; CH_4_ yield −23%; %GEI −36%	Responses may depend on forage type and animal species.	[44]
Increased concentrate proportion or adjusted forage-to-concentrate ratio	DMI +19%; CH_4_ yield −14%; CH_4_ intensity −27%	DMI +23%; CH_4_ yield −26%; CH_4_ intensity −31%	CH_4_ yield −6%; CH_4_ intensity −10%; %GEI +19%	Generally effective in cattle, but sheep responses require caution.	

Note: Data in this table are adapted and summarized mainly from Van Gastelen et al. [44], with additional information from Jonker et al. [45] for corn silage replacement responses in sheep. The values are presented to indicate the general direction and approximate magnitude of responses across ruminant species and dietary strategies. These values should not be interpreted as newly recalculated pooled estimates in the present review. Confidence intervals, standard errors, statistical significance, and heterogeneity statistics were not recalculated; therefore, comparisons among strategies should be interpreted qualitatively and with caution. Abbreviations: NDF, neutral detergent fiber; kd, ruminal degradation rate; ATTD, apparent total-tract digestibility; CH_4_ production, methane production; CH_4_ yield, methane yield; DMI, dry matter intake.

**Table 2:** Comparison of the methane mitigation effects of different additives across ruminant species.

Additive Type	Dairy Cattle	Beef Cattle	Sheep	Evidence Notes	References
3-NOP	CH_4_ production −32.7%; CH_4_ yield −30.9%; CH_4_ intensity −32.6%	CH_4_ production −36.2%; CH_4_ yield −33.2%	Decreased, but quantitative evidence is insufficient	Evidence is strong for dairy and beef cattle, but limited for sheep.	[16,44,46,47]
Nitrate	CH_4_ production −21.5%; CH_4_ yield −15.6%; CH_4_ intensity −20.2%	CH_4_ production −18%; CH_4_ yield −12%; %GEI −14%	CH_4_ production −28%; CH_4_ yield −26%	Generally effective, but the risk of methemoglobinemia should be considered.	[44,48]
Tannin extracts	Inconsistent results; limited number of studies	Inconsistent results; limited number of studies	CH_4_ yield −13%; DMI and CH_4_ production largely unaffected	Responses may depend on tannin source, chemical structure, dose, basal diet, animal species, and physiological state.	[44,49]

Note: Values are adapted and summarized from previously published meta-analyses and evidence syntheses. Data for 3-NOP are mainly derived from Kebreab et al. [16], Van Gastelen et al. [44], de Oliveira et al. [46], and Orzuna-Orzuna et al. [47]. Data for nitrate are mainly derived from Van Gastelen et al. [44] and Dicks et al. [48]. Data for tannin extracts are mainly derived from Van Gastelen et al. [44], with additional support from Berça et al. [49]. These values are intended to indicate approximate response patterns rather than newly recalculated pooled estimates. Confidence intervals, standard errors, and heterogeneity statistics were not recalculated in the present review. Therefore, comparisons among additives and animal species should be interpreted qualitatively and with caution. Abbreviations: 3-NOP, 3-nitrooxypropanol; CH_4_ production, daily methane production; CH_4_ yield, methane production per unit of dry matter intake; CH_4_ intensity, methane production per unit of animal product.

**Table 3:** Effects of harvest maturity of whole-plant corn silage on methane emissions, nutrient composition, and digestibility characteristics in dairy cows.

Item	T25	T28	T32	T40	Trend
DM (%)	25.1	27.7	32	40.3	↑
Starch (g/kg DM)	275	305	356	385	↑
NDF (g/kg DM)	407	394	359	349	↓
Starch kd (/h)	0.098	0.082	0.074	0.059	↓
NDF kd (/h)	0.017	0.013	0.012	0.012	↓
NDF ATTD (%)	53.5	55.2	47.4	44	↓
DM ATTD (%)	70.7	71.9	69.1	66.9	↓
CH_4_ production (g/d)	390	400	386	361	↓
CH_4_ yield (g/kg DMI)	21.7	23	21	20.1	↓

Note: Data are adapted from Hatew et al. [28]. The arrows in the trend column indicate the direction of change with increasing harvest maturity, with “↑” indicating an increasing trend and “↓” indicating a decreasing trend. Abbreviations: DM, dry matter; NDF, neutral detergent fiber; kd, ruminal degradation rate; ATTD, apparent total-tract digestibility; CH_4_ production, methane production; CH_4_ yield, methane yield; DMI, dry matter intake, CH_4_ intensity Methane production per unit of animal product, such as fat- and protein-corrected milk in dairy cow studies.

**Table 4:** Representative nutritional strategies for reducing nitrogen excretion in ruminants.

Strategy	Study Model	Main Responses	References
Reduced dietary crude protein	Lactating dairy cows	Milk urea nitrogen and urinary nitrogen excretion decreased, while production performance was generally maintained when metabolizable protein supply was adequate	[70,72]
Low-protein diet with rumen-protected amino acids	Lactating dairy cows	Responses were inconsistent, suggesting that benefits depend on amino acid balance and dietary context	[73]
Optimization of protein fractions	Sheep	Plasma urea nitrogen and fecal reactive nitrogen emissions decreased; intestinal amino acid transport was improved	[11]
Plant-derived urease inhibitors	In vitro rumen fermentation	Coptisine and epiberberine inhibited ruminal urease activity, reduced ammonia release, and slowed urea degradation	[76,79]
Urease inhibition with microbiome regulation	Cattle rumen microbial system	Allicin promoted urea nitrogen incorporation into microbial amino acids and nucleotides by inhibiting urease activity and reshaping the rumen microbiome	[68]

Note: This table summarizes representative studies discussed in Section 3.1. The results are presented qualitatively and were not recalculated in the present review. Abbreviations: NH_3_, ammonia.

**Table 5:** Regional suitability of nutritional management strategies for ruminants.

Production System	Main Characteristics	Recommended Mitigation Strategies
Temperate intensive systems	High proportion of high-quality silage and compound feed; good consistency of TMR diets; refined feeding management	Precision protein–energy formulation; 3-NOP; nitrate-based inhibition strategies
Tropical–subtropical systems	Higher degree of forage lignification; low digestible fiber content; more pronounced heat stress and seasonal feed fluctuations	Regulation strategies using plant secondary compounds, such as tannins and saponins; physical or biological pretreatment of agricultural by-products
Resource-limited systems	Grazing or semi-grazing predominates; low availability of external additives; limited input levels	Use of local plant-based tannin/saponin resources; pasture improvement; low-cost forage treatment technologies
Grassland–supplementary feeding mixed systems	Grazing shows strong seasonality; diets are more concentrated during the supplementary feeding stage; substrate conditions differ greatly across stages	Stable fermentation regulation strategies during the grazing period; targeted strategies such as nitrates or 3-NOP during the supplementary feeding period

**Table 6:** Strategies not recommended by region.

Region/System Type	Strategies Not Recommended as Priority Options	Main Limiting Factors
Temperate intensive systems	Weak-effect additives with unstable efficacy and relatively high costs	Poor compatibility with highly consistent and precisely formulated diets; limited economic advantage
Tropical–subtropical systems	High-fat diets; high-cost inhibitors that strongly depend on consistent management	Large fluctuations in diet composition, high implementation and management requirements, and insufficient long-term stability
Resource-limited systems	High-cost and supply-chain-dependent strategies, such as 3-NOP	High cost, limited accessibility and continuous supply, and insufficient conditions for widespread adoption
Grazing–supplementation mixed systems	Fixed year-round use of a single mitigation technology	Marked differences in dietary background between grazing and supplementation periods, making it difficult for a single strategy to remain consistently suitable

Note: Table 5 and Table 6 provide qualitative, synthesis-based recommendations rather than quantitatively ranked or region-specific conclusions directly derived from a single study. The recommendations are based on the authors’ interpretation of the evidence discussed in the preceding sections, including studies on plant-derived secondary metabolites [49,52], dietary methane-mitigation strategies and feed additives such as *3-NOP* and nitrate [16,17,44,46,47,48], precision nutrient management [69,87], manure- and soil-interface regulation [92,93,94,95], and regional livestock and pasture mitigation potential [96]. Therefore, the suitability of each strategy should be interpreted according to regional feed resources, production intensity, climate conditions, management capacity, additive accessibility, economic feasibility, and the need to maintain animal performance and rumen stability.

**Table 7:** In vitro methane measurement systems.

Technique Type	Main Applications	Main Advantages	Main Limitations
Pure culture system	Analysis of the metabolism of specific methanogens and screening of inhibitory targets	Clear mechanistic interpretation; fewer variables; suitable for target screening	Difficult to represent the complete rumen microecosystem; limited extrapolation to in vivo conditions
Batch culture system/in vitro gas production	Preliminary screening of diets or additives for methane mitigation	High throughput; low cost; relatively good repeatability	Accumulation of end products; may deviate from in vivo conditions
Continuous culture system	Observation of sustained fermentation responses and microbial adaptation	Relatively stable fermentation status; closer to a continuous fermentation environment	Complex system; relatively low throughput; high maintenance cost
RUSITEC	Medium- to long-term simulation of rumen fermentation and validation of candidate strategies	Can be operated over extended periods; allows quantitative gas collection; relatively good repeatability	Requires high standardization; incomplete maintenance of protozoal populations
DFCC/dual-flow continuous culture	Evaluation of fermentation, degradation, and nitrogen metabolism responses	Controllable solid and liquid dilution rates; suitable for mechanistic validation	Complex maintenance; considerable variation among laboratories

Note: Information in this table was synthesized from previous methodological reviews and studies [7,98,99,100,101,102,103]. Abbreviations: *RUSITEC*, rumen simulation technique; *DFCC*, dual-flow continuous culture.

## Data Availability

No new data were created or analyzed in this study.

## Abbreviations

The following abbreviations are used in this manuscript

Abbreviation	Full Term
3-NOP	3-nitrooxypropanol
ATTD	Apparent total-tract digestibility
CAZymes	Carbohydrate-active enzymes
CH_4_	Methane
CO_2_	Carbon dioxide
DFCC	Dual-flow continuous culture
DM	Dry matter
DMI	Dry matter intake
EC	Eddy covariance
EPR	Electron paramagnetic resonance
FPCM	Fat- and protein-corrected milk
GEI	Gross energy intake
H_2_	Hydrogen
kd	Ruminal degradation rate
LCA	Life cycle assessment
MCR	Methyl-coenzyme M reductase
mcrA	Gene encoding the alpha subunit of methyl-coenzyme M reductase
NADH	Nicotinamide adenine dinucleotide, reduced form
NASEM	National Academies of Sciences, Engineering, and Medicine
NDF	Neutral detergent fiber
NH_3_	Ammonia
NH_3_-N	Ammonia nitrogen
N_2_O	Nitrous oxide
NRC	National Research Council
OM	Organic matter
PAM	Polyacrylamide
pH	Potential of hydrogen
RUSITEC	Rumen simulation technique
TMR	Total mixed ration
VFA	Volatile fatty acid
HB	Hydrogenobody
FDP	Flavodiiron oxygen reductase
Hyd	Hydrogenase
